# Supplementary material for: Clinical features and novel technologies for prehospital detection of intracerebral haemorrhage: a scoping review protocol
Source: BMJ Open. 2023 May 3;13(5):e070228. doi: 10.1136/bmjopen-2022-070228 (PMC10163533; doi:10.1136/bmjopen-2022-070228)
Supplement: Author's manuscript [file bmjopen-2022-070228.draft_revisions.pdf]

BMJ Open

BMJ Open is committed to open peer review. As part of this commitment we make the peer review history of every article we publish publicly available.

When an article is published we post the peer reviewers' comments and the authors' responses online. We also post the versions of the paper that were used during peer review. These are the versions that the peer review comments apply to.

The versions of the paper that follow are the versions that were submitted during the peer review process. They are not the versions of record or the final published versions. They should not be cited or distributed as the published version of this manuscript.

BMJ Open is an open access journal and the full, final, typeset and author-corrected version of record of the manuscript is available on our site with no access controls, subscription charges or pay-per-view fees (http://bmjopen.bmj.com).

If you have any questions on BMJ Open's open peer review process please email
info.bmjopen@bmj.com

BMJ Open

Clinical features and novel technologies for pre-hospital detection of intracerebral haemorrhage: a scoping review protocol

Journal:	*BMJ Open*
Manuscript ID	bmjopen-2022-070228
Article Type:	Protocol
Date Submitted by the Author:	17-Nov-2022
Complete List of Authors:	Almubayyidh, Mohammed; The University of Manchester, Division of Cardiovascular Sciences; King Saud University, Department of Aviation and Marine<br>Alghamdi, Ibrahim; The University of Manchester, Division of Cardiovascular Sciences; King Khalid University, Department of Emergency Medical Services<br>Parry-Jones, Adrian; The University of Manchester, Division of Cardiovascular Sciences; Northern Care Alliance NHS Foundation Trust, Manhcester Centre for Clinical Neurosciences<br>Jenkins, David; The University of Manchester, Division of Informatics, Imaging and Data Science
Keywords:	Stroke < NEUROLOGY, STROKE MEDICINE, ACCIDENT & EMERGENCY MEDICINE

SCHOLARONE™
Manuscripts

Clinical features and novel technologies for pre-hospital detection of intracerebral haemorrhage: a scoping review protocol

Mohammed Almubayyidh[1,2*], Ibrahim Alghamdi[1,3], Adrian Parry-Jones[1,4], David Jenkins[5]

Affiliations

[1]Division of Cardiovascular Sciences, School of Medical Sciences, Faculty of Biology, Medicine and Health, The University of Manchester, Manchester, M13 9PL, UK

[2]Department of Aviation and Marine, Prince Sultan Bin Abdulaziz College for Emergency Medical Services, King Saud University, Riyadh, KSA

[3]Department of Emergency Medical Services, College of Applied Medical Sciences - Khamis Mushait, King Khalid University, Abha, KSA

[4]Manhcester Centre for Clinical Neurosciences, Northern Care Alliance NHS Foundation Trust, Stott Lane, Salford, M6 8HD, UK

[5]Division of Informatics, Imaging and Data Science, School of Health Sciences, Faculty of Biology, Medicine and Health, The University of Manchester, Manchester, M13 9PL, UK

*Corresponding Author:

Mohammed Almubayyidh, mohammed.almubayyidh@postgrad.manchester.ac.uk, Division of Cardiovascular Sciences, School of Medical Sciences, Faculty of Biology, Medicine and Health, The University of Manchester, Manchester, M13 9PL, UK

Abstract word count: 301

Text word count: 2025

For peer review only

ABSTRACT

Introduction

The detection of intracerebral haemorrhage (ICH) in the pre-hospital setting without conventional imaging technology would allow widespread and cost-effective early treatment to reduce haematoma expansion and improve patient outcomes. Although, ICH and ischaemic stroke share many clinical features, some may help in distinguishing ICH from other suspected stroke patients. In combination with clinical features, novel technologies may improve diagnosis further. This scoping review aims to first identify the early, distinguishing clinical features of ICH and then identify novel portable imaging technologies that may enhance differentiation of ICH from other suspected strokes. Where appropriate and feasible, meta-analyses will be performed.

Methods

The scoping review will follow the recommendations of the Joanna Briggs Institute Methodology for Scoping Reviews as well as the Preferred Reporting Items for Systematic Reviews and Meta-Analyses extension for Scoping Reviews checklist. A systematic search will be conducted using MEDLINE (Ovid), EMBASE (Ovid) and CENTRAL (Ovid). EndNote reference management software will be used to remove duplicate entries. Two independent reviewers will screen titles, abstracts and full-text reports according to prespecified eligibility criteria using the Rayyan Qatar Computing Research Institute (QCRI) software. One reviewer will screen all titles, abstracts and full-text reports of potentially eligible studies, whilst the other reviewer will independently screen at least 20% of all titles, abstracts, and full-text reports. Conflicts will be resolved through discussion or by consulting a third reviewer. Results will be tabulated in accordance with the scoping review's objectives along with a narrative discussion.

For peer review only

Ethics and dissemination

Ethical approval is not required for this review, as it will only include published literature. The results will be published in an open-access, peer-reviewed journal, presented at scientific conferences, and form part of a PhD thesis. We expect the findings to contribute to future research into the early detection of ICH in suspected stroke patients.

Keywords: Protocol, Intracerebral hemorrhage, Stroke, Differential diagnosis, Detection, Clinical features, Prehospital, Emergency, Imaging, Technology.

Strengths and limitations of this study

- This scoping review will address an important knowledge gap about the early detection of ICH in suspected stroke patients to help pre-hospital personnel during the field assessment, treatment and destination triage of these patients.
- No time of publication restrictions will be applied to comprehensively map the area of interest.
- The review will be limited to English language publications only.
- A formal risk of bias assessment of the included studies will not be performed, as this is beyond the purpose of a scoping review.

For peer review only

INTRODUCTION

Early detection of intracerebral haemorrhage

Intracerebral haemorrhage (ICH) has the highest mortality rate of any stroke subtype [1]. The one-year case fatality of ICH is estimated to be around 50%, with nearly half of those deaths occurring within 72 hours of onset, predominantly due to neurological complications [2,3]. The risk is greatest in this early stage, as the haematoma can expand during the first few hours after the onset of symptoms. A delay in interventions for ICH or other strokes may lead to poor patient outcomes [2]. As treatment options are highly time-dependent, the early recognition of symptoms and the rapid delivery of interventions are crucial for effective management.

The majority of strokes occur at home, meaning that pre-hospital personnel are typically the first to make clinical contact with suspected stroke patients [4]. These personnel play a crucial role in the early triage and management of these patients. The initial diagnosis of stroke is based on clinical presentation and stroke recognition instruments, followed by neuroimaging confirmation in the hospital. Brain imaging using computed tomography (CT) and magnetic resonance imaging (MRI) remains the gold standard for detecting stroke subtypes and accurate recognition of stroke subtypes prior to imaging is not felt to be possible [5]. The current American Heart Association and American Stroke Association guidelines state "No existing clinical decision scale can differentiate ICH from other diseases with high sensitivity or specificity in the absence of neuroimaging", but a systematic review of the literature was not presented and novel technologies were not discussed [6].

For peer review only

The accurate recognition of stroke may be challenging for pre-hospital personnel due to the heterogeneity of clinical presentations, time constraints and the lack of accurate diagnostic technology in this setting [7]. Stroke treatment has become increasingly complex with critically time-sensitive procedures, such as intravenous thrombolysis, mechanical thrombectomy or neurosurgical operations only available in specialised hospitals (e.g., hyper-acute stroke unit) [8]. It is thus desirable to not only differentiate stroke from non-stroke but to identify subtypes (ICH vs ischaemic stroke) and eligibility for interventions, ensuring patients are transported directly to a centre with the capability to deliver the appropriate interventions. Pre-hospital recognition of patients with ICH will facilitate the very early delivery of treatments to prevent haematoma expansion, such as intensive blood pressure reduction, anticoagulant reversal and haemostatic agents, when they may be far more effective [9,10].

Clinical features and emerging technologies to detect and classify ICH

Many clinical features of ICH are also features of other stroke types though some have been described that may help differentiate ICH from other suspected stroke patients [11]. For example, patients with spontaneous ICH tend to be younger than those with ischaemic stroke [11]. Also, the presence of severe hypertension (systolic blood pressure ≥180 mm Hg), headache, vomiting, seizure and decreased consciousness level is more common on admission in patients with ICH; meanwhile, a history of transient neurological deficits, hyperlipidaemia and atrial fibrillation is more common in patients with ischaemic stroke [12]. On average, the neurological deficits in patients with ICH tend to be more severe than they are in patients with ischaemic stroke [13]. A combination of these clinical features may be useful to predict ICH in suspected stroke patients.

Recent literature has proposed new approaches that could assist pre-hospital personnel recognise stroke subtypes, opening new ways to diagnose stroke and provide treatment earlier [7]. These include, but are not limited to, the development of new stroke screening tools, blood biomarkers technologies and new imaging modalities [7,8,11,13-16]. Many of these advances are still in the early stages of development; therefore, further

For peer review only

investigation is required to determine whether they can detect stroke sufficiently and differentiate between stroke types, particularly within the first few hours of symptoms onset, and their potential use in pre-hospital care.

Objectives

The purpose of this scoping review is to summarise and describe the current state of knowledge regarding the early identification of ICH in suspected stroke patients. First, we aim to identify early clinical features that can help distinguish ICH patients from other stroke types and non-stroke diagnoses. Second, we aim to explore portable imaging technologies that can be used by pre-hospital personnel to detect ICH. Finally, we will determine whether meta-analyses are appropriate and feasible based on the homogeneity of findings.

METHODS

Protocol design

This study will follow the guidelines of the Joanna Briggs Institute (JBI) Methodology for Scoping Reviews [17,18]. The Preferred Reporting Items for Systematic Reviews and Meta-Analyses extension for Scoping Reviews (PRISMA-ScR) checklist will be followed to ensure that all suggested items are reported (Appendix 1) [19]. This protocol will be reported using the PRISMA-ScR checklist and JBI guidelines.

Inclusion/exclusion criteria:

The population, concept, context (PCC) framework is used to establish eligibility criteria. Studies will be selected according to the following criteria:

Population

Included studies will describe adult patients (aged ≥16 years) with suspected stroke or intracranial haemorrhage confirmed by either CT or MRI scans. We will exclude studies of children (aged <16) from this review due to their different presentations, diagnostic

For peer review only

challenges, risk factors and recurrence risk [20]. Additionally, we will exclude studies that combined the clinical features of spontaneous ICH patients with other aetiologies, such as subarachnoid haemorrhage, without describing them separately.

Concept

We will first examine the concept of distinguishing spontaneous ICH from other stroke types and non-stroke diagnoses using early clinical features collected at the initial pre-hospital assessment or within the first 24 hours of symptoms onset at hospital admission. Secondly, we will explore the possibility of detecting ICH using portable imaging devices. Studies should report the clinical characteristics of their included patients, including but not limited to, demographic data, signs and symptoms at onset, vital signs (e.g., blood pressure, heart rate and blood glucose) and past medical histories. Studies that evaluate novel portable imaging devices will be required to report the accuracy of diagnosis (e.g., sensitivity, specificity and/or discrimination) after testing the device on ICH patients. Where appropriate, we will evaluate the applicability of utilising the evidence identified in pre-hospital care. Studies that report the clinical features of patients after 24 hours of symptoms onset or only examined ICH patients without comparing them to other stroke or non-stroke diagnoses will be excluded from this review. Also, we will exclude studies that examine advances in conventional detection modalities, such as CT or MRI, or tested the technology only on phantom or animal models. Also, those studies that only used a machine-learning algorithm to detect stroke without a portable imaging device will be excluded.

Context

The context of this scoping review is the pre-hospital and in-hospital care setting. It will include sources that report on healthcare professionals, including physicians, paramedics, nurses and others who provide patient care in a variety of settings, such as emergency departments, intensive care units and ambulance facilities. Primary care studies will not be included in this review. Further, there will be no restrictions on the country of study, ethnicity, gender or socioeconomic status.

For peer review only

Sources of information and search strategy

In this scoping review, we will include data from primary research studies. The search will be limited to human studies published in English. Conference abstracts, commentaries, surveys, case reports, animal studies and articles in languages other than English will be excluded. The search strategy was developed based on specific keywords that were combined using the Boolean operators ("AND", "OR"), which can be found in Appendix 2. This search strategy will be applied to MEDLINE via Ovid, EMBASE via Ovid and CENTRAL via Ovid, from the date of inception to August 2022. Finally, we will review the reference lists of the included articles to identify other additional studies, thus minimising the likelihood of missing any eligible publication.

Study records and selection process

Following the database search, all identified studies will be imported into EndNote and Rayyan Qatar Computing Research Institute (QCRI) software to remove duplicates, as well as to facilitate screening and collaboration. The selection of studies for inclusion in the review will be conducted by two independent reviewers in two stages. Initially, the titles and abstracts will be screened, and then a full-text review will be conducted. The first reviewer (MA) will screen the titles and abstracts to remove studies that do not meet the predefined inclusion/exclusion criteria before retrieving the full-text of eligible studies. The second reviewer (IA) will independently screen a random selection of at least 20% of the titles and abstracts. Any discrepancies about the suitability of any of the papers will be resolved through discussion, or by consulting a third reviewer (APJ or DAJ) as required. The first reviewer (MA) will then review the full-text reports against the inclusion and exclusion criteria. The second reviewer (IA) will independently screen at least 20% of the full-text reports as well, and any discrepancies will be resolved through discussion, or by consulting a third reviewer (APJ or DAJ) as required. We will record the reasons for exclusion after the full-text assessment of works that do not meet the inclusion criteria. Detailed information about the search and the study inclusion process will be presented in the final scoping review report with a PRISMA-ScR flow chart [19].

For peer review only

Data extraction process and items

The general characteristics of the included studies will be summarised using basic publication data (e.g., authors, publication year, country, research design, study environment), study population information (e.g., sample size, stroke types, clinical features collected), details of the detection device (e.g., technology used, diagnostic accuracy, time to results, potential limitations, size/portability) and key findings of the studies. The summary of results will be presented in different tables in accordance with the scoping review's objectives. The tabulated results will be accompanied by a narrative description of the evidence. Furthermore, the results will be described in terms of how they address the objectives of this review.

Data synthesis and quality assessment of studies.

The analysis of ICH detection methods relies on the data obtained from each study. As one of the objectives of this scoping review is to establish whether there are sufficient data available to facilitate a subsequent systematic review and meta-analyses, no quantitative analysis will be carried out. Furthermore, since there is no expectation for a bias assessment on the PRISMA-ScR checklist [19], we will not conduct a formal assessment of the risk of bias of the included studies.

PATIENT AND PUBLIC INVOLVEMENT

In this scoping review, the data collected are based on previously published studies, so neither the patients nor the general public will be involved.

ETHICS AND DISSEMINATION

The results of this review will be published in an open-access, peer-reviewed journal, and the findings will be presented at scientific conferences and will be part of a PhD thesis. We expect that the study findings will be useful in future to develop research into the early detection of ICH in suspected stroke patients by pre-hospital personnel, and to help

For peer review only

them make field assessments and decisions about the treatment and destination triage of these patients. This scoping review will only investigate published literature; therefore, ethical approval is not required.

CONCLUSION

As treatment options are highly time-dependent, the effective management of ICH patients requires the early recognition of clinical features, delivery of appropriate interventions and correct transport decisions to a hospital with neurosurgical care for eligible patients. Although suspected stroke patients may have similar or overlapping clinical features, some early findings may suggest the presence of ICH. To facilitate the diagnosis, several new technologies and approaches have been proposed to enhance the pre-hospital care of suspected stroke. Our study will inform future research by mapping the literature to help pre-hospital personnel distinguish ICH from other suspected stroke patients to improve the field assessment, treatment and destination triage to the appropriate level of care.

AUTHOR CONTRIBUTIONS

MA is the primary author of this document under the supervision of APJ and DAJ. DAJ conceptualised the idea for the review. MA designed the search strategy and drafted the protocol paper, incorporating the suggestions put forward by APJ, DAJ and IA. All authors critically reviewed the protocol and approved the final manuscript before final submission for peer review.

FUNDING STATEMENT

This study is funded by King Saud University, Saudi Arabia, through the Saudi Arabian Cultural Bureau in the United Kingdom.

For peer review only

CONFLICTS OF INTEREST

None declared.

REFERENCES

1. Sahni R, Weinberger J. Management of intracerebral hemorrhage. Vasc Health Risk Manag. 2007;3(5):701-9.

2. Ishfaq M, Goyal N, Pandhi A, et al. Prehospital and Emergency Department Management of Intracerebral Hemorrhage: Concepts and Customs. 2018. p. 1-16.

3. Hu YZ, Wang JW, Luo BY. Epidemiological and clinical characteristics of 266 cases of intracerebral hemorrhage in Hangzhou, China. J Zhejiang Univ Sci B. 2013;14(6):496-504.

4. Massaro AR, Sacco RL, Scaff M, et al. Clinical discriminators between acute brain hemorrhage and infarction: a practical score for early patient identification. Arq Neuropsiquiatr. 2002;60(2-a):185-91.

5. Smith EE, Kent DM, Bulsara KR, et al. Accuracy of Prediction Instruments for Diagnosing Large Vessel Occlusion in Individuals With Suspected Stroke: A Systematic Review for the 2018 Guidelines for the Early Management of Patients With Acute Ischemic Stroke. Stroke. 2018;49(3):e111-e22.

6. Greenberg SM, Ziai WC, Cordonnier C, et al. 2022 Guideline for the Management of Patients With Spontaneous Intracerebral Hemorrhage: A Guideline From the American Heart Association/American Stroke Association. Stroke. 2022;53(7):e282-e361.

7. Lumley HA, Flynn D, Shaw L, et al. A scoping review of pre-hospital technology to assist ambulance personnel with patient diagnosis or stratification during the emergency assessment of suspected stroke. BMC Emerg Med. 2020;20(1):30.

8. Geisler F, Wesirow M, Ebinger M, et al. Probability assessment of intracerebral hemorrhage in prehospital emergency patients. Neurol Res Pract. 2021;3(1):1-.

9. Rodriguez-Luna D, Rodriguez-Villatoro N, Juega JM, et al. Prehospital Systolic Blood Pressure Is Related to Intracerebral Hemorrhage Volume on Admission. Stroke. 2018;49(1):204-6.

For peer review only

10. Yassi N, Zhao H, Churilov L, et al. Tranexamic acid for intracerebral haemorrhage within 2 hours of onset: protocol of a phase II randomised placebo-controlled double-blind multicentre trial. Stroke and Vascular Neurology. 2022;7(2):158.

11. Jin HQ, Wang JC, Sun YA, et al. Prehospital Identification of Stroke Subtypes in Chinese Rural Areas. Chin Med J (Engl). 2016;129(9):1041-6.

12. Besson G, Robert C, Hommel M, et al. Is It Clinically Possible to Distinguish Nonhemorrhagic Infarct From Hemorrhagic Stroke? Stroke. 1995;26(7):1205-9.

13. Yamashita S, Kimura K, Iguchi Y, et al. Kurashiki Prehospital Stroke Subtyping Score (KP3S) as a means of distinguishing ischemic from hemorrhagic stroke in emergency medical services. Eur Neurol. 2011;65(4):233-8.

14. Uchida K, Yoshimura S, Sakakibara F, et al. Simplified Prehospital Prediction Rule to Estimate the Likelihood of 4 Types of Stroke: The 7-Item Japan Urgent Stroke Triage (JUST-7) Score. Prehosp Emerg Care. 2021;25(4):465-74.

15. Cooley SR, Zhao H, Campbell BCV, et al. Mobile Stroke Units Facilitate Prehospital Management of Intracerebral Hemorrhage. Stroke. 2021;52(10):3163-6.

16. Mattila OS, Ashton NJ, Blennow K, et al. Ultra-Early Differential Diagnosis of Acute Cerebral Ischemia and Hemorrhagic Stroke by Measuring the Prehospital Release Rate of GFAP. Clinical Chemistry. 2021;67(10):1361-72.

17. Peters M, Godfrey C, Khalil H, et al. 2017 Guidance for the Conduct of JBI Scoping Reviews. 2017.

18. Peters MDJ, Marnie C, Colquhoun H, et al. Scoping reviews: reinforcing and advancing the methodology and application. Syst Rev. 2021;10(1):263.

19. Tricco AC, Lillie E, Zarin W, et al. PRISMA Extension for Scoping Reviews (PRISMA-ScR): Checklist and Explanation. Ann Intern Med. 2018;169(7):467-73.

20. Jordan LC, Hillis AE. Challenges in the diagnosis and treatment of pediatric stroke. Nat Rev Neurol. 2011;7(4):199-208.

For peer review only

Appendix 1. Preferred Reporting Items for Systematic reviews and Meta-Analyses extension for Scoping Reviews (PRISMA-ScR) Checklist

SECTION	ITEM	PRISMA-ScR CHECKLIST ITEM	REPORTED ON PAGE #
**TITLE**			
Title	1	Identify the report as a scoping review.	1
**ABSTRACT**			
Structured summary	2	Provide a structured summary that includes (as applicable): background, objectives, eligibility criteria, sources of evidence, charting methods, results, and conclusions that relate to the review questions and objectives.	2-3
**INTRODUCTION**			
Rationale	3	Describe the rationale for the review in the context of what is already known. Explain why the review questions/objectives lend themselves to a scoping review approach.	4-6
Objectives	4	Provide an explicit statement of the questions and objectives being addressed with reference to their key elements (e.g., population or participants, concepts, and context) or other relevant key elements used to conceptualize the review questions and/or objectives.	6-7
**METHODS**			
Protocol and registration	5	Indicate whether a review protocol exists; state if and where it can be accessed (e.g., a Web address); and if available, provide registration information, including the registration number.	N/A
Eligibility criteria	6	Specify characteristics of the sources of evidence used as eligibility criteria (e.g., years considered, language, and publication status), and provide a rationale.	6-8
Information sources*	7	Describe all information sources in the search (e.g., databases with dates of coverage and contact with authors to identify additional sources), as well as the date the most recent search was executed.	7-8
Search	8	Present the full electronic search strategy for at least 1 database, including any limits used, such that it could be repeated.	Appendix 2
Selection of sources of evidence†	9	State the process for selecting sources of evidence (i.e., screening and eligibility) included in the scoping review.	8
Data charting process‡	10	Describe the methods of charting data from the included sources of evidence (e.g., calibrated forms or forms that have been tested by the team before their use, and whether data charting was done independently or in duplicate) and any processes for obtaining and confirming data from investigators.	8-9
Data items	11	List and define all variables for which data were sought and any assumptions and simplifications made.	Appendix 2
Critical appraisal of individual sources of evidence§	12	If done, provide a rationale for conducting a critical appraisal of included sources of evidence; describe the methods used and how this information was used in any data synthesis (if appropriate).	N/A
Synthesis of results	13	Describe the methods of handling and summarizing the data that were charted.	8-9

St. Michael's
Inspired Care. Inspiring Science.

For peer review only - http://bmjopen.bmj.com/site/about/guidelines.xhtml

SECTION	ITEM	PRISMA-ScR CHECKLIST ITEM	REPORTED ON PAGE #
**RESULTS**			
Selection of sources of evidence	14	Give numbers of sources of evidence screened, assessed for eligibility, and included in the review, with reasons for exclusions at each stage, ideally using a flow diagram.	N/A
Characteristics of sources of evidence	15	For each source of evidence, present characteristics for which data were charted and provide the citations.	N/A
Critical appraisal within sources of evidence	16	If done, present data on critical appraisal of included sources of evidence (see item 12).	N/A
Results of individual sources of evidence	17	For each included source of evidence, present the relevant data that were charted that relate to the review questions and objectives.	N/A
Synthesis of results	18	Summarize and/or present the charting results as they relate to the review questions and objectives.	N/A
**DISCUSSION**			
Summary of evidence	19	Summarize the main results (including an overview of concepts, themes, and types of evidence available), link to the review questions and objectives, and consider the relevance to key groups.	N/A
Limitations	20	Discuss the limitations of the scoping review process.	N/A
Conclusions	21	Provide a general interpretation of the results with respect to the review questions and objectives, as well as potential implications and/or next steps.	N/A
**FUNDING**			
Funding	22	Describe sources of funding for the included sources of evidence, as well as sources of funding for the scoping review. Describe the role of the funders of the scoping review.	10

JBI = Joanna Briggs Institute; PRISMA-ScR = Preferred Reporting Items for Systematic reviews and Meta-Analyses extension for Scoping Reviews.
* Where *sources of evidence* (see second footnote) are compiled from, such as bibliographic databases, social media platforms, and Web sites.
† A more inclusive/heterogeneous term used to account for the different types of evidence or data sources (e.g., quantitative and/or qualitative research, expert opinion, and policy documents) that may be eligible in a scoping review as opposed to only studies. This is not to be confused with *information sources* (see first footnote).
‡ The frameworks by Arksey and O'Malley (6) and Levac and colleagues (7) and the JBI guidance (4, 5) refer to the process of data extraction in a scoping review as data charting.
§ The process of systematically examining research evidence to assess its validity, results, and relevance before using it to inform a decision. This term is used for items 12 and 19 instead of "risk of bias" (which is more applicable to systematic reviews of interventions) to include and acknowledge the various sources of evidence that may be used in a scoping review (e.g., quantitative and/or qualitative research, expert opinion, and policy document).

From: Tricco AC, Lillie E, Zarin W, O'Brien KK, Colquhoun H, Levac D, et al. PRISMA Extension for Scoping Reviews (PRISMAScR): Checklist and Explanation. Ann Intern Med. 2018;169:467–473. doi: 10.7326/M18-0850.

For peer review only - http://bmjopen.bmj.com/site/about/guidelines.xhtml

Appendix 2. Search strategies for the scoping review

1- The search strategy for clinical features

Database searched: MEDLINE (Ovid)

Date of covering: From inception to August 2022

PCC framework: Population, Concept, Context

#	Query
1	exp Brain Ischemia/ or exp Stroke/ or exp Hemorrhagic Stroke/ or exp Cerebral Hemorrhage/ or exp Intracranial Hemorrhages/
2	Intracerebral hemorrhage.mp.
3	1 or 2
4	exp cerebrovascular accident/di
5	exp stroke/di
6	4 or 5
7	(recogni$ or identi$ or strati$ or different$ or probabilit$).mp.
8	3 and 7
9	6 or 8
10	exp Emergency Medical Services/ or Paramedic*.mp. or ambulance.mp. or triage.mp. or prehospital.mp. or pre hospital.mp. or EMS.mp. or emergency medical service*.mp.
11	Air Ambulances/
12	Emergency Service, Hospital/
13	Emergency Medicine/
14	Clinical feature.mp.
15	Stroke Score.mp.
16	Clinical differentiation.mp.
17	10 or 11 or 12 or 13 or 14 or 15 or 16
18	9 and 17
19	limit 18 to english language

For peer review only

Database searched: EMBASE (Ovid)

Date of covering: From inception to August 2022

PCC framework: Population, Concept, Context

#	Query
1	exp Brain Ischemia/ or exp Stroke/ or exp Hemorrhagic Stroke/ or exp Cerebral Hemorrhage/ or exp Intracranial Hemorrhages/
2	Intracerebral hemorrhage.mp.
3	1 or 2
4	exp cerebrovascular accident/di [Diagnosis]
5	exp stroke/di
6	4 or 5
7	(recogni$ or identi$ or strati$ or distinguish$ or probabilit$).mp.
8	3 and 7
9	6 or 8
10	exp Emergency Medical Services/ or Paramedic*.mp. or ambulance.mp. or triage.mp. or prehospital.mp. or pre hospital.mp. or EMS.mp. or emergency medical service*.mp. or out-of-hospital.mp.
11	Air Ambulances/
12	Emergency Service, Hospital/
13	Stroke Score.mp.
14	Clinical differentiation.mp.
15	10 or 11 or 12 or 13 or 14
16	9 and 15
17	limit 16 to (human and english language)

For peer review only

Database searched: CENTRAL (Ovid)

Date of covering: From inception to August 2022

PCC framework: Population, Concept, Context

#	Query
1	exp Brain Ischemia/ or exp Stroke/ or exp Hemorrhagic Stroke/ or exp Cerebral Hemorrhage/ or exp Intracranial Hemorrhages/
2	Intracerebral hemorrhage.mp.
3	1 or 2
4	exp cerebrovascular accident/di
5	exp stroke/di
6	4 or 5
7	(recogni$ or identi$ or strati$ or different$ or probabilit$).mp.
8	3 and 7
9	6 or 8
10	exp Emergency Medical Services/ or Paramedic*.mp. or ambulance.mp. or triage.mp. or prehospital.mp. or pre hospital.mp. or EMS.mp. or emergency medical service*.mp.
11	Air Ambulances/
12	Emergency Service, Hospital/
13	Emergency Medicine/
14	Clinical feature.mp.
15	Stroke Score.mp.
16	Clinical differentiation.mp.
17	10 or 11 or 12 or 13 or 14 or 15 or 16
18	9 and 17
19	limit 18 to english language

For peer review only

2- The search strategy for portable imaging devices

Database searched: MEDLINE (Ovid)

Date of covering: From inception to August 2022

PCC framework: Population, Concept, Context

#	Query
1	exp Brain Ischemia/ or exp Stroke/ or exp Hemorrhagic Stroke/ or exp Cerebral Hemorrhage/ or exp Intracranial Hemorrhages/
2	Intracerebral hemorrhage.mp.
3	1 or 2
4	(recogni$ or identi$ or strati$ or diagnos$ or detect$).mp.
5	exp Emergency Medical Services/ or Paramedic*.mp. or ambulance.mp. or triage.mp. or prehospital.mp. or pre hospital.mp. or EMS.mp. or emergency medical service*.mp.
6	Air Ambulances/
7	Emergency Service, Hospital/
8	(technology or spectroscopy or ultrasound or diagnostic imaging or imaging or transcranial doppler or radiofrequency or microwave or electroencephalography or device).mp.
9	5 or 6 or 7
10	3 and 4 and 8 and 9
11	limit 10 to (english language and humans)

For peer review only

Database searched: EMBASE (Ovid)

Date of covering: From inception to August 2022

PCC framework: Population, Concept, Context

#	Query
1	exp Brain Ischemia/ or exp Stroke/ or exp Hemorrhagic Stroke/ or exp Cerebral Hemorrhage/ or exp Intracranial Hemorrhages/
2	Intracerebral hemorrhage.mp.
3	1 or 2
4	(recogni$ or identi$ or strati$ or diagnos$ or detect$).mp.
5	exp Emergency Medical Services/ or Paramedic*.mp. or ambulance.mp. or triage.mp. or prehospital.mp. or pre hospital.mp. or EMS.mp. or emergency medical service*.mp.
6	Air Ambulances/
7	Emergency Service, Hospital/
8	(technology or spectroscopy or ultrasound or diagnostic imaging or imaging or transcranial doppler or radiofrequency or microwave or electroencephalography or device).mp.
9	5 or 6 or 7
10	3 and 4 and 8 and 9
11	limit 10 to (english language and humans)

For peer review only

Database searched: CENTRAL (Ovid)

Date of covering: From inception to August 2022

PCC framework: Population, Concept, Context

#	Query
1	exp Brain Ischemia/ or exp Stroke/ or exp Hemorrhagic Stroke/ or exp Cerebral Hemorrhage/ or exp Intracranial Hemorrhages/
2	Intracerebral hemorrhage.mp.
3	1 or 2
4	(recogni$ or identi$ or strati$ or diagnos$ or detect$).mp.
5	exp Emergency Medical Services/ or Paramedic*.mp. or ambulance.mp. or triage.mp. or prehospital.mp. or pre hospital.mp. or EMS.mp. or emergency medical service*.mp.
6	Air Ambulances/
7	Emergency Service, Hospital/
8	(technology or spectroscopy or ultrasound or diagnostic imaging or imaging or transcranial doppler or radiofrequency or microwave or electroencephalography or device).mp.
9	5 or 6 or 7
10	3 and 4 and 8 and 9
11	limit 10 to (english language and humans)

For peer review only

BMJ Open

Clinical features and novel technologies for pre-hospital detection of intracerebral haemorrhage: a scoping review protocol

Journal:	*BMJ Open*
Manuscript ID	bmjopen-2022-070228.R1
Article Type:	Protocol
Date Submitted by the Author:	16-Mar-2023
Complete List of Authors:	Almubayyidh, Mohammed; The University of Manchester, Division of Cardiovascular Sciences; King Saud University, Department of Aviation and Marine<br>Alghamdi, Ibrahim; The University of Manchester, Division of Cardiovascular Sciences; King Khalid University, Department of Emergency Medical Services<br>Parry-Jones, Adrian; The University of Manchester, Division of Cardiovascular Sciences; Northern Care Alliance NHS Foundation Trust, Manhcester Centre for Clinical Neurosciences<br>Jenkins, David; The University of Manchester, Division of Informatics, Imaging and Data Science
**Primary Subject Heading**:	Emergency medicine
Secondary Subject Heading:	Emergency medicine
Keywords:	Stroke < NEUROLOGY, STROKE MEDICINE, ACCIDENT & EMERGENCY MEDICINE

SCHOLARONE™
Manuscripts

Clinical features and novel technologies for pre-hospital detection of intracerebral haemorrhage: a scoping review protocol

Mohammed Almubayyidh[1,2*], Ibrahim Alghamdi[1,3], Adrian Parry-Jones[1,4], David Jenkins[5]

Affiliations

[1]Division of Cardiovascular Sciences, School of Medical Sciences, Faculty of Biology, Medicine and Health, The University of Manchester, Manchester, M13 9PL, UK

[2]Department of Aviation and Marine, Prince Sultan Bin Abdulaziz College for Emergency Medical Services, King Saud University, Riyadh, KSA

[3]Department of Emergency Medical Services, College of Applied Medical Sciences - Khamis Mushait, King Khalid University, Abha, KSA

[4]Manhcester Centre for Clinical Neurosciences, Northern Care Alliance NHS Foundation Trust, Stott Lane, Salford, M6 8HD, UK

[5]Division of Informatics, Imaging and Data Science, School of Health Sciences, Faculty of Biology, Medicine and Health, The University of Manchester, Manchester, M13 9PL, UK

*Corresponding Author:

Mohammed Almubayyidh, mohammed.almubayyidh@postgrad.manchester.ac.uk, Division of Cardiovascular Sciences, School of Medical Sciences, Faculty of Biology, Medicine and Health, The University of Manchester, Manchester, M13 9PL, UK

Abstract word count: 296

Text word count: 2077

For peer review only

ABSTRACT

Introduction

The detection of intracerebral haemorrhage (ICH) in the pre-hospital setting without conventional imaging technology might allow early treatment to reduce haematoma expansion and improve patient outcomes. Although ICH and ischaemic stroke share many clinical features, some may help in distinguishing ICH from other suspected stroke patients. In combination with clinical features, novel technologies may improve diagnosis further. This scoping review aims to first identify the early, distinguishing clinical features of ICH and then identify novel portable technologies that may enhance differentiation of ICH from other suspected strokes. Where appropriate and feasible, meta-analyses will be performed.

Methods

The scoping review will follow the recommendations of the Joanna Briggs Institute Methodology for Scoping Reviews as well as the Preferred Reporting Items for Systematic Reviews and Meta-Analyses extension for Scoping Reviews checklist. A systematic search will be conducted using MEDLINE (Ovid), EMBASE (Ovid) and CENTRAL (Ovid). EndNote reference management software will be used to remove duplicate entries. Two independent reviewers will screen titles, abstracts and full-text reports according to prespecified eligibility criteria using the Rayyan Qatar Computing Research Institute (QCRI) software. One reviewer will screen all titles, abstracts and full-text reports of potentially eligible studies, whilst the other reviewer will independently screen at least 20% of all titles, abstracts, and full-text reports. Conflicts will be resolved through discussion or by consulting a third reviewer. Results will be tabulated in accordance with the scoping review's objectives along with a narrative discussion.

Ethics and dissemination

Ethical approval is not required for this review, as it will only include published literature. The results will be published in an open-access, peer-reviewed journal, presented at scientific conferences, and form part of a PhD thesis. We expect the findings to contribute to future research into the early detection of ICH in suspected stroke patients.

For peer review only

Keywords: Protocol, Intracerebral hemorrhage, Stroke, Differential diagnosis, Detection, Clinical features, Prehospital, Emergency, Imaging, Technology.

Strengths and limitations of this study

- No time of publication restrictions will be applied to comprehensively map the area of interest.
- The review will be limited to English language publications only.
- A formal risk of bias assessment will not be performed.

For peer review only

INTRODUCTION

Early detection of intracerebral haemorrhage

Intracerebral haemorrhage (ICH) has the highest mortality rate of any stroke subtype [1]. The one-year case fatality of ICH is estimated to be around 50%, with nearly half of those deaths occurring within 72 hours of onset, predominantly due to neurological complications [2,3]. The risk is greatest in this early stage, as the haematoma can expand during the first few hours after the onset of symptoms [4]. Consequently, ongoing clinical trials of different treatment approaches are targeting the very early stages of the disease process, with the aim to improve outcomes for patients with ICH by reducing haematoma expansion [5-7]. A delay in interventions for ICH may lead to poor patient outcomes [2]. As treatment options appear highly time-dependent [8], the early recognition of symptoms and the rapid delivery of interventions are crucial for effective management.

The majority of strokes occur at home, meaning that pre-hospital personnel are typically the first to make clinical contact with suspected stroke patients [9]. These personnel play a crucial role in the early triage and management of these patients. The initial diagnosis of stroke is based on clinical presentation and stroke recognition instruments, followed by neuroimaging confirmation in the hospital. Brain imaging using computed tomography (CT) and magnetic resonance imaging (MRI) remains the gold standard for detecting stroke subtypes and accurate recognition of stroke subtypes prior to imaging is not felt to be possible [10]. The current American Heart Association and American Stroke Association guidelines state “No existing clinical decision scale can differentiate ICH from other diseases with high sensitivity or specificity in the absence of neuroimaging”, but a systematic review of the literature was not presented and novel technologies were not discussed [11].

For peer review only

The accurate recognition of stroke may be challenging for pre-hospital personnel due to the heterogeneity of clinical presentations, time constraints and the lack of accurate diagnostic technology in this setting [12]. Stroke treatment has become increasingly complex with critically time-sensitive procedures, such as intravenous thrombolysis, mechanical thrombectomy or neurosurgical operations only available in specialised hospitals (e.g., hyper-acute stroke unit) [13]. It is thus desirable to not only differentiate stroke from non-stroke but to identify subtypes (ICH vs ischaemic stroke) and eligibility for interventions, ensuring patients are transported directly to a centre with the capability to deliver the appropriate interventions. Pre-hospital recognition of patients with ICH will facilitate the very early delivery of treatments to prevent haematoma expansion, such as intensive blood pressure reduction, anticoagulant reversal and haemostatic agents, which may potentially improve outcomes [7,14]. Nevertheless, it is important to note that such interventions must be thoroughly studied in adequately powered and well-designed clinical trials to determine their efficacy and safety. The ability to diagnose ICH in the ambulance easily and inexpensively would facilitate such clinical trials.

Clinical features and emerging technologies to detect and classify ICH

Many clinical features of ICH are also features of other stroke types though some have been described that may help differentiate ICH from other suspected stroke patients [15]. For example, patients with spontaneous ICH tend to be younger than those with ischaemic stroke [15]. Also, the presence of hypertension and headache is more common on admission in patients with ICH; meanwhile, a history of transient neurological deficits, hyperlipidaemia and atrial fibrillation is more common in patients with ischaemic stroke [16]. These features were used to develop clinical prediction rules to distinguish ICH from ischaemic stroke [16,17]. Nevertheless, the diagnostic accuracy of these rules are low and may not be applicable to patients presenting within a few hours of the onset of symptoms or during the pre-hospital phase [15,18].

Recent literature has proposed new approaches that could assist pre-hospital personnel recognise stroke subtypes, opening new ways to diagnose stroke and provide treatment earlier [12]. These include, but are not limited to, the development of new stroke screening tools, blood biomarkers technologies and new imaging modalities [12,13,19-26]. Many of these advances are still in the early stages of development; therefore, further investigation is required to determine whether they can detect stroke sufficiently and differentiate between stroke types, particularly within the first few hours of symptoms onset, and their potential use in pre-hospital care.

For peer review only

Objectives

The purpose of this scoping review is to summarise and describe the current state of knowledge regarding the early identification of ICH in suspected stroke patients. First, we aim to identify early clinical features that can help distinguish ICH patients from other stroke types and non-stroke diagnoses. Second, we aim to explore portable technologies that can be used by pre-hospital personnel to detect ICH. Finally, we will determine whether meta-analyses are appropriate and feasible based on the homogeneity of findings.

METHODS

Protocol design

This study will follow the guidelines of the Joanna Briggs Institute (JBI) Methodology for Scoping Reviews [27,28]. The Preferred Reporting Items for Systematic Reviews and Meta-Analyses extension for Scoping Reviews (PRISMA-ScR) checklist will be followed to ensure that all suggested items are reported (Appendix 1) [29]. This protocol will be reported using the PRISMA-ScR checklist and JBI guidelines.

Inclusion/exclusion criteria:

The population, concept, context (PCC) framework is used to establish eligibility criteria, as suggested by the JBI [27,28]. Table 1 summarises the PCC framework of this protocol.

PCC	Included	Excluded
**Population**	• Studies of adult patients (aged ≥16 years) with suspected stroke or intracranial haemorrhage	• Studies of children (aged <16 years)<br>• Studies combining the clinical features of ICH with those of other aetiologies

For peer review only

**Concept**	• Studies reported early clinical features of suspected stroke patients, collected at pre-hospital assessment or within 24 hours of symptoms onset at hospital admission<br>• Studies tested portable technologies to detect ICH	• Studies reported clinical features of patients after 24 hours of symptoms onset; or if they investigated clinical features associated with ICH without comparing them against other suspected stroke cases<br>• Studies that did not report on the diagnostic performance of the tested technology; or examined advances in conventional detection modalities; or that tested the technology only on phantoms or animal models
**Context**	• Pre-hospital and in-hospital studies	• Primary care studies

Table 1. Inclusion and exclusion criteria

Population

Included studies will describe adult patients (aged ≥16 years) with suspected stroke or intracranial haemorrhage confirmed by either CT or MRI scans. We will exclude studies of children (aged <16 years) from this review due to their different presentations, diagnostic challenges, risk factors and recurrence risk [30]. Additionally, we will exclude studies that combined the clinical features of spontaneous ICH patients with other aetiologies, such as subarachnoid haemorrhage, without describing them separately.

Concept

We will first examine the concept of distinguishing spontaneous ICH from other stroke types and non-stroke diagnoses using early clinical features collected at the initial pre-hospital assessment or within the first 24 hours of symptoms onset at hospital admission. Secondly, we will explore the possibility of detecting ICH using portable devices. Studies should report the clinical characteristics of their included patients, including but not limited to, demographic data, signs and symptoms at onset, vital signs (e.g., blood pressure, heart rate and blood glucose) and past medical histories. Studies that evaluate novel portable devices will be required to report the accuracy of diagnosis (e.g., sensitivity, specificity and/or discrimination) after testing the device on ICH patients. Where appropriate, we will evaluate the applicability of utilising the evidence identified in pre-hospital care. Studies that report the clinical features of patients after 24 hours of symptoms onset or only examined ICH patients' clinical features without comparing them to

For peer review only

other stroke or non-stroke diagnoses will be excluded from this review. Also, we will exclude studies that examine advances in conventional detection modalities, such as CT or MRI, or tested the technology only on phantom or animal models.

Context

The context of this scoping review is the pre-hospital and in-hospital care setting. It will include sources that report on healthcare professionals, including physicians, paramedics, nurses and others who provide patient care in a variety of settings, such as emergency departments, intensive care units and ambulance facilities. Primary care studies will not be included in this review. Further, there will be no restrictions on the country of study, ethnicity, gender or socioeconomic status.

Sources of information and search strategy

In this scoping review, we will include data from primary research studies. The search will be limited to human studies, and for resource reasons, will be restricted to studies published in English. Conference abstracts, commentaries, surveys, case reports, animal studies and articles in languages other than English will be excluded. The search strategy was developed based on specific keywords that were combined using the Boolean operators ("AND", "OR"), which can be found in Appendix 2. This search strategy will be applied to MEDLINE via Ovid, EMBASE via Ovid and CENTRAL via Ovid, from the date of inception to August 2022. Finally, we will review the reference lists of the included articles to identify other additional studies, thus minimising the likelihood of missing any eligible publication.

Study records and selection process

Following the database search, all identified studies will be imported into EndNote and Rayyan Qatar Computing Research Institute (QCRI) software to remove duplicates, as well as to facilitate screening and collaboration. The selection of studies for inclusion in the review will be conducted by two independent reviewers in two stages. Initially, the titles and abstracts will be screened, and then a full-text review will be conducted. The first reviewer (MA) will screen the titles and abstracts to remove studies that do not meet the predefined inclusion/exclusion criteria before retrieving the full-text of eligible studies. The second reviewer (IA) will independently screen a random selection of at least 20% of the titles and abstracts. Any discrepancies about the suitability

For peer review only

of any of the papers will be resolved through discussion, or by consulting a third reviewer (APJ or DAJ) as required. The first reviewer (MA) will then review the full-text reports against the inclusion and exclusion criteria. The second reviewer (IA) will independently screen at least 20% of the full-text reports as well, and any discrepancies will be resolved through discussion, or by consulting a third reviewer (APJ or DAJ) as required. We will record the reasons for exclusion after the full-text assessment of works that do not meet the inclusion criteria. Detailed information about the search and the study inclusion process will be presented in the final scoping review report with a PRISMA-ScR flow chart [29].

Data extraction process and items

The general characteristics of the included studies will be summarised using basic publication data (e.g., authors, publication year, country, research design, study environment), study population information (e.g., sample size, stroke types, clinical features collected), details of the detection device (e.g., technology used, diagnostic accuracy, time to results, potential limitations) and key findings of the studies. The summary of results will be presented in different tables in accordance with the scoping review's objectives. The tabulated results will be accompanied by a narrative description of the evidence. Furthermore, the results will be described in terms of how they address the objectives of this review.

Data synthesis and quality assessment of studies

The analysis of ICH detection methods relies on the data obtained from each study. Given that this is a scoping review, a narrative synthesis will be used to summarise the findings of the included studies. Furthermore, since there is no expectation for a bias assessment on the PRISMA-ScR checklist [29], we will not conduct a formal assessment of the risk of bias of the included studies.

PATIENT AND PUBLIC INVOLVEMENT

In this scoping review, the data collected are based on previously published studies, so neither the patients nor the general public will be involved.

For peer review only

ETHICS AND DISSEMINATION

The results of this review will be published in an open-access, peer-reviewed journal, and the findings will be presented at scientific conferences and will be part of a PhD thesis. We expect that the study findings will be useful in future to develop research into the early detection of ICH in suspected stroke patients by pre-hospital personnel, and to help them make field assessments and decisions about the treatment and destination triage of these patients. This scoping review will only investigate published literature; therefore, ethical approval is not required.

CONCLUSION

As treatment options appear highly time-dependent, the effective management of ICH patients requires the early recognition of clinical features, delivery of appropriate interventions and correct transport decisions to a hospital with neurosurgical care for eligible patients. Although suspected stroke patients may have similar or overlapping clinical features, some early findings may suggest the presence of ICH. To facilitate the diagnosis, several new technologies and approaches have been proposed to enhance the pre-hospital care of suspected stroke. Our study will inform future research by mapping the literature to help pre-hospital personnel distinguish ICH from other suspected stroke patients to improve the field assessment, treatment and destination triage to the appropriate level of care.

AUTHOR CONTRIBUTIONS

MA is the primary author of this document under the supervision of APJ and DAJ. DAJ conceptualised the idea for the review. MA designed the search strategy and drafted the protocol paper, incorporating the suggestions put forward by APJ, DAJ and IA. All authors critically reviewed the protocol and approved the final manuscript before final submission for peer review.

FUNDING STATEMENT

This study is funded by King Saud University, Saudi Arabia, through the Saudi Arabian Cultural Bureau in the United Kingdom (FG-492106).

For peer review only

CONFLICTS OF INTEREST

None declared.

REFERENCES

1. Sahni R, Weinberger J. Management of intracerebral hemorrhage. Vasc Health Risk Manag. 2007;3(5):701-9.

2. Ishfaq M, Goyal N, Pandhi A, et al. Prehospital and Emergency Department Management of Intracerebral Hemorrhage: Concepts and Customs. 2018. p. 1-16.

3. Hu YZ, Wang JW, Luo BY. Epidemiological and clinical characteristics of 266 cases of intracerebral hemorrhage in Hangzhou, China. J Zhejiang Univ Sci B. 2013;14(6):496-504.

4. Al-Shahi Salman R, Frantzias J, Lee RJ, et al. Absolute risk and predictors of the growth of acute spontaneous intracerebral haemorrhage: a systematic review and meta-analysis of individual patient data. Lancet Neurol. 2018;17(10):885-94.

5. Naidech AM, Grotta J, Elm J, et al. Recombinant factor VIIa for hemorrhagic stroke treatment at earliest possible time (FASTEST): Protocol for a phase III, double-blind, randomized, placebo-controlled trial. Int J Stroke. 2021:806-9.

6. Song L, Chen C, Chen X, et al. INTEnsive ambulance-delivered blood pressure Reduction in hyper-ACute stroke Trial (INTERACT4): study protocol for a randomized controlled trial. Trials. 2021;22(1):885.

7. Yassi N, Zhao H, Churilov L, et al. Tranexamic acid for intracerebral haemorrhage within 2 hours of onset: protocol of a phase II randomised placebo-controlled double-blind multicentre trial. Stroke Vasc Neurol. 2022;7(2):158-65.

8. Sandset EC, Anderson CS, Bath PM, et al. European Stroke Organisation (ESO) guidelines on blood pressure management in acute ischaemic stroke and intracerebral haemorrhage. Eur Stroke J. 2021;6(2):XLVIII-LXXXIX.

9. Massaro AR, Sacco RL, Scaff M, et al. Clinical discriminators between acute brain hemorrhage and infarction: a practical score for early patient identification. Arq Neuropsiquiatr. 2002;60(2-a):185-91.

10. Smith EE, Kent DM, Bulsara KR, et al. Accuracy of Prediction Instruments for Diagnosing Large Vessel Occlusion in Individuals With Suspected Stroke: A Systematic Review for the 2018

For peer review only

Guidelines for the Early Management of Patients With Acute Ischemic Stroke. Stroke. 2018;49(3):e111-e22.

11. Greenberg SM, Ziai WC, Cordonnier C, et al. 2022 Guideline for the Management of Patients With Spontaneous Intracerebral Hemorrhage: A Guideline From the American Heart Association/American Stroke Association. Stroke. 2022;53(7):e282-e361.

12. Lumley HA, Flynn D, Shaw L, et al. A scoping review of pre-hospital technology to assist ambulance personnel with patient diagnosis or stratification during the emergency assessment of suspected stroke. BMC Emerg Med. 2020;20(1):30.

13. Geisler F, Wesirow M, Ebinger M, et al. Probability assessment of intracerebral hemorrhage in prehospital emergency patients. Neurol Res Pract. 2021;3(1):1.

14. Rodriguez-Luna D, Rodriguez-Villatoro N, Juega JM, et al. Prehospital Systolic Blood Pressure Is Related to Intracerebral Hemorrhage Volume on Admission. Stroke. 2018;49(1):204-6.

15. Runchey S, McGee S. Does this patient have a hemorrhagic stroke? Clinical findings distinguishing hemorrhagic stroke from ischemic stroke. JAMA. 2010;303(22):2280-6.

16. Besson G, Robert C, Hommel M, et al. Is It Clinically Possible to Distinguish Nonhemorrhagic Infarct From Hemorrhagic Stroke? Stroke. 1995;26(7):1205-9.

17. Poungvarin N, Viriyavejakul A, Komontri C. Siriraj stroke score and validation study to distinguish supratentorial intracerebral haemorrhage from infarction. BMJ. 1991;302(6792):1565-7.

18. Badam P, Solao V, Pai M, et al. Poor accuracy of the Siriraj and Guy's hospital stroke scores in distinguishing haemorrhagic from ischaemic stroke in a rural, tertiary care hospital. Natl Med J India. 2003;16(1):8-12.

19. Yamashita S, Kimura K, Iguchi Y, et al. Kurashiki Prehospital Stroke Subtyping Score (KP3S) as a means of distinguishing ischemic from hemorrhagic stroke in emergency medical services. Eur Neurol. 2011;65(4):233-8.

20. Jin HQ, Wang JC, Sun YA, et al. Prehospital Identification of Stroke Subtypes in Chinese Rural Areas. Chin Med J (Engl). 2016;129(9):1041-6.

21. Uchida K, Yoshimura S, Hiyama N, et al. Clinical Prediction Rules to Classify Types of Stroke at Prehospital Stage. Stroke. 2018;49(8):1820-7.

22. Uchida K, Yoshimura S, Sakakibara F, et al. Simplified Prehospital Prediction Rule to Estimate the Likelihood of 4 Types of Stroke: The 7-Item Japan Urgent Stroke Triage (JUST-7) Score. Prehosp Emerg Care. 2021;25(4):465-74.

For peer review only

23. Mattila OS, Ashton NJ, Blennow K, et al. Ultra-Early Differential Diagnosis of Acute Cerebral Ischemia and Hemorrhagic Stroke by Measuring the Prehospital Release Rate of GFAP. Clin Chem. 2021;67(10):1361-72.

24. Cooley SR, Zhao H, Campbell BCV, et al. Mobile Stroke Units Facilitate Prehospital Management of Intracerebral Hemorrhage. Stroke. 2021;52(10):3163-6.

25. Persson M, Fhager A, Trefná HD, et al. Microwave-based stroke diagnosis making global prehospital thrombolytic treatment possible. IEEE Trans Biomed Eng. 2014;61(11):2806-17.

26. Antipova D, Eadie L, Makin S, et al. The use of transcranial ultrasound and clinical assessment to diagnose ischaemic stroke due to large vessel occlusion in remote and rural areas. PLoS One. 2020;15(10):e0239653.

27. Peters M, Godfrey C, Khalil H, et al. 2017 Guidance for the Conduct of JBI Scoping Reviews. 2017.

28. Peters MDJ, Marnie C, Colquhoun H, et al. Scoping reviews: reinforcing and advancing the methodology and application. Syst Rev. 2021;10(1):263.

29. Tricco AC, Lillie E, Zarin W, et al. PRISMA Extension for Scoping Reviews (PRISMA-ScR): Checklist and Explanation. Ann Intern Med. 2018;169(7):467-73.

30. Jordan LC, Hillis AE. Challenges in the diagnosis and treatment of pediatric stroke. Nat Rev Neurol. 2011;7(4):199-208.

For peer review only

Appendix 1. Preferred Reporting Items for Systematic reviews and Meta-Analyses extension for Scoping Reviews (PRISMA-ScR) Checklist

SECTION	ITEM	PRISMA-ScR CHECKLIST ITEM	REPORTED ON PAGE #
**TITLE**			
Title	1	Identify the report as a scoping review.	1
**ABSTRACT**			
Structured summary	2	Provide a structured summary that includes (as applicable): background, objectives, eligibility criteria, sources of evidence, charting methods, results, and conclusions that relate to the review questions and objectives.	2
**INTRODUCTION**			
Rationale	3	Describe the rationale for the review in the context of what is already known. Explain why the review questions/objectives lend themselves to a scoping review approach.	4-6
Objectives	4	Provide an explicit statement of the questions and objectives being addressed with reference to their key elements (e.g., population or participants, concepts, and context) or other relevant key elements used to conceptualize the review questions and/or objectives.	6-7
**METHODS**			
Protocol and registration	5	Indicate whether a review protocol exists; state if and where it can be accessed (e.g., a Web address); and if available, provide registration information, including the registration number.	N/A
Eligibility criteria	6	Specify characteristics of the sources of evidence used as eligibility criteria (e.g., years considered, language, and publication status), and provide a rationale.	6-8
Information sources*	7	Describe all information sources in the search (e.g., databases with dates of coverage and contact with authors to identify additional sources), as well as the date the most recent search was executed.	8
Search	8	Present the full electronic search strategy for at least 1 database, including any limits used, such that it could be repeated.	Appendix 2
Selection of sources of evidence†	9	State the process for selecting sources of evidence (i.e., screening and eligibility) included in the scoping review.	8
Data charting process‡	10	Describe the methods of charting data from the included sources of evidence (e.g., calibrated forms or forms that have been tested by the team before their use, and whether data charting was done independently or in duplicate) and any processes for obtaining and confirming data from investigators.	8-9
Data items	11	List and define all variables for which data were sought and any assumptions and simplifications made.	Appendix 2
Critical appraisal of individual sources of evidence§	12	If done, provide a rationale for conducting a critical appraisal of included sources of evidence; describe the methods used and how this information was used in any data synthesis (if appropriate).	N/A
Synthesis of results	13	Describe the methods of handling and summarizing the data that were charted.	8-9

For peer review only

St. Michael's
Inspired Care.
Inspiring Science.

For peer review only - http://bmjopen.bmj.com/site/about/guidelines.xhtml

SECTION	ITEM	PRISMA-ScR CHECKLIST ITEM	REPORTED ON PAGE #
**RESULTS**			
Selection of sources of evidence	14	Give numbers of sources of evidence screened, assessed for eligibility, and included in the review, with reasons for exclusions at each stage, ideally using a flow diagram.	N/A
Characteristics of sources of evidence	15	For each source of evidence, present characteristics for which data were charted and provide the citations.	N/A
Critical appraisal within sources of evidence	16	If done, present data on critical appraisal of included sources of evidence (see item 12).	N/A
Results of individual sources of evidence	17	For each included source of evidence, present the relevant data that were charted that relate to the review questions and objectives.	N/A
Synthesis of results	18	Summarize and/or present the charting results as they relate to the review questions and objectives.	N/A
**DISCUSSION**			
Summary of evidence	19	Summarize the main results (including an overview of concepts, themes, and types of evidence available), link to the review questions and objectives, and consider the relevance to key groups.	N/A
Limitations	20	Discuss the limitations of the scoping review process.	N/A
Conclusions	21	Provide a general interpretation of the results with respect to the review questions and objectives, as well as potential implications and/or next steps.	N/A
**FUNDING**			
Funding	22	Describe sources of funding for the included sources of evidence, as well as sources of funding for the scoping review. Describe the role of the funders of the scoping review.	10

JBI = Joanna Briggs Institute; PRISMA-ScR = Preferred Reporting Items for Systematic reviews and Meta-Analyses extension for Scoping Reviews.

* Where *sources of evidence* (see second footnote) are compiled from, such as bibliographic databases, social media platforms, and Web sites.

† A more inclusive/heterogeneous term used to account for the different types of evidence or data sources (e.g., quantitative and/or qualitative research, expert opinion, and policy documents) that may be eligible in a scoping review as opposed to only studies. This is not to be confused with *information sources* (see first footnote).

‡ The frameworks by Arksey and O'Malley (6) and Levac and colleagues (7) and the JBI guidance (4, 5) refer to the process of data extraction in a scoping review as data charting.

§ The process of systematically examining research evidence to assess its validity, results, and relevance before using it to inform a decision. This term is used for items 12 and 19 instead of "risk of bias" (which is more applicable to systematic reviews of interventions) to include and acknowledge the various sources of evidence that may be used in a scoping review (e.g., quantitative and/or qualitative research, expert opinion, and policy document).

From: Tricco AC, Lillie E, Zarin W, O'Brien KK, Colquhoun H, Levac D, et al. PRISMA Extension for Scoping Reviews (PRISMAScR): Checklist and Explanation. Ann Intern Med. 2018;169:467–473. doi: 10.7326/M18-0850.

For peer review only - http://bmjopen.bmj.com/site/about/guidelines.xhtml

Appendix 2. Search strategies for the scoping review

1- The search strategy for clinical features

Database searched: MEDLINE (Ovid)

Date of covering: From inception to August 2022

PCC framework: Population, Concept, Context

#	Query
1	exp Brain Ischemia/ or exp Stroke/ or exp Hemorrhagic Stroke/ or exp Cerebral Hemorrhage/ or exp Intracranial Hemorrhages/
2	Intracerebral hemorrhage.mp.
3	1 or 2
4	exp cerebrovascular accident/di
5	exp stroke/di
6	4 or 5
7	(recogni$ or identi$ or strati$ or different$ or probabilit$).mp.
8	3 and 7
9	6 or 8
10	exp Emergency Medical Services/ or Paramedic*.mp. or ambulance.mp. or triage.mp. or prehospital.mp. or pre hospital.mp. or EMS.mp. or emergency medical service*.mp.
11	Air Ambulances/
12	Emergency Service, Hospital/
13	Emergency Medicine/
14	Clinical feature.mp.
15	Stroke Score.mp.
16	Clinical differentiation.mp.
17	10 or 11 or 12 or 13 or 14 or 15 or 16
18	9 and 17
19	limit 18 to english language

For peer review only

Database searched: EMBASE (Ovid)

Date of covering: From inception to August 2022

PCC framework: Population, Concept, Context

#	Query
1	exp Brain Ischemia/ or exp Stroke/ or exp Hemorrhagic Stroke/ or exp Cerebral Hemorrhage/ or exp Intracranial Hemorrhages/
2	Intracerebral hemorrhage.mp.
3	1 or 2
4	exp cerebrovascular accident/di [Diagnosis]
5	exp stroke/di
6	4 or 5
7	(recogni$ or identi$ or strati$ or distinguish$ or probabilit$).mp.
8	3 and 7
9	6 or 8
10	exp Emergency Medical Services/ or Paramedic*.mp. or ambulance.mp. or triage.mp. or prehospital.mp. or pre hospital.mp. or EMS.mp. or emergency medical service*.mp. or out-of-hospital.mp.
11	Air Ambulances/
12	Emergency Service, Hospital/
13	Stroke Score.mp.
14	Clinical differentiation.mp.
15	10 or 11 or 12 or 13 or 14
16	9 and 15
17	limit 16 to (human and english language)

For peer review only

Database searched: CENTRAL (Ovid)

Date of covering: From inception to August 2022

PCC framework: Population, Concept, Context

#	Query
1	exp Brain Ischemia/ or exp Stroke/ or exp Hemorrhagic Stroke/ or exp Cerebral Hemorrhage/ or exp Intracranial Hemorrhages/
2	Intracerebral hemorrhage.mp.
3	1 or 2
4	exp cerebrovascular accident/di
5	exp stroke/di
6	4 or 5
7	(recogni$ or identi$ or strati$ or different$ or probabilit$).mp.
8	3 and 7
9	6 or 8
10	exp Emergency Medical Services/ or Paramedic*.mp. or ambulance.mp. or triage.mp. or prehospital.mp. or pre hospital.mp. or EMS.mp. or emergency medical service*.mp.
11	Air Ambulances/
12	Emergency Service, Hospital/
13	Emergency Medicine/
14	Clinical feature.mp.
15	Stroke Score.mp.
16	Clinical differentiation.mp.
17	10 or 11 or 12 or 13 or 14 or 15 or 16
18	9 and 17
19	limit 18 to english language

For peer review only

2- The search strategy for portable devices

Database searched: MEDLINE (Ovid)

Date of covering: From inception to August 2022

PCC framework: Population, Concept, Context

#	Query
1	exp Brain Ischemia/ or exp Stroke/ or exp Hemorrhagic Stroke/ or exp Cerebral Hemorrhage/ or exp Intracranial Hemorrhages/
2	Intracerebral hemorrhage.mp.
3	1 or 2
4	(recogni$ or identi$ or strati$ or diagnos$ or detect$).mp.
5	exp Emergency Medical Services/ or Paramedic*.mp. or ambulance.mp. or triage.mp. or prehospital.mp. or pre hospital.mp. or EMS.mp. or emergency medical service*.mp.
6	Air Ambulances/
7	Emergency Service, Hospital/
8	(technology or spectroscopy or ultrasound or diagnostic imaging or imaging or transcranial doppler or radiofrequency or microwave or electroencephalography or device).mp.
9	5 or 6 or 7
10	3 and 4 and 8 and 9
11	limit 10 to (english language and humans)

For peer review only

Database searched: EMBASE (Ovid)

Date of covering: From inception to August 2022

PCC framework: Population, Concept, Context

#	Query
1	exp Brain Ischemia/ or exp Stroke/ or exp Hemorrhagic Stroke/ or exp Cerebral Hemorrhage/ or exp Intracranial Hemorrhages/
2	Intracerebral hemorrhage.mp.
3	1 or 2
4	(recogni$ or identi$ or strati$ or diagnos$ or detect$).mp.
5	exp Emergency Medical Services/ or Paramedic*.mp. or ambulance.mp. or triage.mp. or prehospital.mp. or pre hospital.mp. or EMS.mp. or emergency medical service*.mp.
6	Air Ambulances/
7	Emergency Service, Hospital/
8	(technology or spectroscopy or ultrasound or diagnostic imaging or imaging or transcranial doppler or radiofrequency or microwave or electroencephalography or device).mp.
9	5 or 6 or 7
10	3 and 4 and 8 and 9
11	limit 10 to (english language and humans)

For peer review only

Database searched: CENTRAL (Ovid)

Date of covering: From inception to August 2022

PCC framework: Population, Concept, Context

#	Query
1	exp Brain Ischemia/ or exp Stroke/ or exp Hemorrhagic Stroke/ or exp Cerebral Hemorrhage/ or exp Intracranial Hemorrhages/
2	Intracerebral hemorrhage.mp.
3	1 or 2
4	(recogni$ or identi$ or strati$ or diagnos$ or detect$).mp.
5	exp Emergency Medical Services/ or Paramedic*.mp. or ambulance.mp. or triage.mp. or prehospital.mp. or pre hospital.mp. or EMS.mp. or emergency medical service*.mp.
6	Air Ambulances/
7	Emergency Service, Hospital/
8	(technology or spectroscopy or ultrasound or diagnostic imaging or imaging or transcranial doppler or radiofrequency or microwave or electroencephalography or device).mp.
9	5 or 6 or 7
10	3 and 4 and 8 and 9
11	limit 10 to (english language and humans)

For peer review only
